# Altered CD4+/CD8+ T-cell ratio in splenocytes of human T-cell leukemia virus type I Tax transgenic mice with inflammatory arthropathy

**DOI:** 10.1186/1742-4690-8-S1-A2

**Published:** 2011-06-06

**Authors:** Takeo Ohsugi, Toshio Kumasaka

**Affiliations:** 1 Institute of Resource Development and Analysis, Kumamoto University, 2-2-1 Honjo, Kumamoto 860-0811, Japan; 2 Department of Pathology, Japanese Red Cross Medical Center, 4-1-22 Hiroo, Shibuya-ku, Tokyo 150-8935, Japan

## 

Human T-cell leukemia virus type I (HTLV-1) infection in human causes an aggressive malignancy, adult T-cell leukemia (ATL), as well as inflammatory diseases such as HTLV-1-associated myelopathy/tropical spastic paraparesis (HAM/TSP). A transgenic mouse model expressing Tax in mature thymocytes and peripheral T lymphocytes caused mature T cell leukemia resembling ATL. We also found that inflammatory arthropathy develops among Tax-expressing mice without leukemia. The pathological findings of arthropathy in Tax transgenic mice were similar to those seen in human rheumatoid arthritis or mouse models of rheumatoid arthritis, with synovial proliferation and a positive rheumatoid factor. No group differences between control mice and arthropathic mice were found in the proportion of T and B cells in spleens. The arthropathic mice showed a significantly decreased proportion of splenic CD4+ T cells, whereas the proportion of splenic CD8+ T cells was increased. The Th17 cells were not detected in arthropathic mice. Regulatory T cells (CD4+CD25+Foxp3+) were significantly decreased and CD8+ T cells that expressed the chemokine receptor CCR4 (CD8+CCR4+) were significantly increased in arthropathic Tax transgenic mice. The expression of tax mRNA was strong in the spleen and joints of arthropathic mice, with a 40-fold increase compared with healthy-transgenic mice.Tax transgenic mice develop rheumatoid-like arthritis; however, splenic T-cell subsets in these mice was completely different from other commonly used animal models of rheumatoid arthritis. The crucial T-cell subsets in these mice appear to resemble those in HAM/TSP patients rather than those in rheumatoid arthritis patients.

